# Evaluation of the effects of locally applied rosuvastatin on bone formation in a three-dimensional reconstruction rabbit xenograft model

**DOI:** 10.3906/sag-2011-109

**Published:** 2021-08-24

**Authors:** Taha ÖZER, Alper AKTAŞ, Canseda AVAĞ, Ayşegül FIRAT, Mert OCAK, Fevziye Figen KAYMAZ, Hakan Hamdi ÇELİK

**Affiliations:** 1 Department of Oral and Maxillofacial Surgery, Faculty of Dentistry, Hacettepe University, Ankara, Turkey; 2 Department of Anatomy, Faculty of Medicine, Hacettepe University, Ankara, Turkey; 3 Vocational School of Health, Ankara University, Ankara, Turkey; 4 Department of Histology and Embryology, Faculty of Medicine, Hacettepe University, Ankara, Turkey

**Keywords:** Rosuvastatin, xenografting, bone transplantation, osteogenesis, HMG-CoA reductase inhibitors

## Abstract

**Background/aim:**

Guided bone regeneration (GBR) is commonly performed to repair bone defects, and rigid occlusive titanium barriers play a vital role in bone formation in regions with no prior bone tissue. The statin, rosuvastatin (RSV), strongly affects bone apposition when applied locally. Here, we aimed to evaluate the anabolic effects of locally applied RSV with a xenograft placed on rabbit calvaria.

**Materials and methods:**

Two rigid occlusive titanium caps were used in 16 rabbits after decorticating the calvarial bone. In the control group, the area under the cap was filled with a xenograft, while in the RSV group, a xenograft in combination with RSV (1 mg) was used. In both groups, at 6 and 12 weeks, new bone, residual graft, soft tissue areas, and histological and radiological bone volume were evaluated.

**Results:**

At 12 weeks, histologically, the RSV group exhibited superior new bone proportion values, and radiologically, new bone and total bone volume in the RSV group were significantly higher than in the control group (p < 0.05); there were no significant differences at 6 weeks (p > 0.05).

**Conclusion:**

According to our results, RSV applied locally under a titanium barrier on an area to be repaired with bone grafts increases new bone and total bone volume.

## 1. Introduction

Guided bone regeneration (GBR) is commonly used to repair bone defects associated with pathological lesions or to increase bone volume before dental implant treatment [1]. The barrier membranes that are used in GBR are vitally important for appropriate bone formation. These membranes prevent soft tissue from growing toward the inside of the defect, and they protect the defect site until bone tissue development is complete. The membranes used in GBR need to have several characteristics in order to ensure maximum bone regeneration. The most important of these are biocompatibility, stiffness to maintain the defect cavity, ability to prevent epithelial cell migration, and permeability to ensure that the newly formed tissues are fed [2]. However, another view suggests that a similar amount of bone development can be achieved with the use of impermeable titanium barriers as that achieved on using permeable membranes [3]. The only drawback of titanium barriers is that they require a second surgery because they are not self-resorbable, despite which in many studies, guided tissue regeneration using titanium barriers has been successfully performed for bone regeneration in rabbits [4,5].

Statins, which are 3-hydroxy-3-methylglutaryl coenzyme-A (HMG-CoA) reductase inhibitors, were first developed to treat patients suffering from hyperlipidemia and hypercholesterolemia, and they were observed to have positive effects in osteopathic patients as well [6]. Recently, statins were also shown to have a positive role in bone formation as they modulate inflammation and increase osteogenesis and angiogenesis [7,8]. Statins increase the release of important osteoanabolic and angiogenetic factors, such as bone morphogenetic protein (BMP-2) and vascular endothelial growth factor (VEGF), [7]. Rosuvastatin (RSV) is a lipid-lowering agent that is used to prevent cardiovascular disorders. In addition, it has a longer half-life and produces stronger effects than simvastatin and atorvastatin [9]. RSV is also known to have pleiotropic effects, including bone stimulation, increased vascularization, and antiinflammatory effects. Following oral administration, statins are rapidly absorbed; however, their systemic bioavailability is low owing to their widespread first-pass metabolism in the liver [10]. Therefore, the oral administration of statins is known to be ineffective in healing bone defects. Several studies have revealed their positive effects on bone formation through oral and subcutaneous administrations, and recent research has been primarily aimed at uncovering the optimal dosage and formulation of locally administered RSV [11,12]. However, the effects of locally administered RSV in GBR procedures in areas with no prior bone formation still remain unclear.

Therefore, the aim of this study was to histologically and radiologically evaluate the effects of RSV administered locally together with a xenograft placed under a titanium barrier at the bone augmentation site in a rabbit calvaria.

## 2. Materials and methods

### 2.1. Experimental model

This study protocol was performed in accordance with National Institutes of Health (NIH) ARRIVE guidelines for the care and use of laboratory animals and independently reviewed and approved by the Animal Experiments Local Ethics Committee of Hacettepe University on 29 May 2018 under the identification number, 2018/27-05. Sixteen male New Zealand rabbits were used in this study. The experimental animals were housed in cages at standardized room temperature, humidity, ventilation, and fluorescent light (imitating a light/dark cycle of 12 h each) settings for a period of 1 week before the study was commenced in order for them to adapt to the environmental and climatic conditions. The study was designed such that each of the experimental animals provided samples for both of the study groups listed below (from the right side of the calvaria for one group and from the left side of the calvaria for the other).

- RSV + xenograft group (RSV)
- Xenograft/control group (C)

For the evaluation, each group was further divided based on two healing period time frames, 6 weeks and 12 weeks. Care was taken to administer the RSV group treatment on the left side of the calvaria and the C group treatment on the right side of the calvaria in each animal.

### 2.2. Surgical procedure

**Figure 1:**
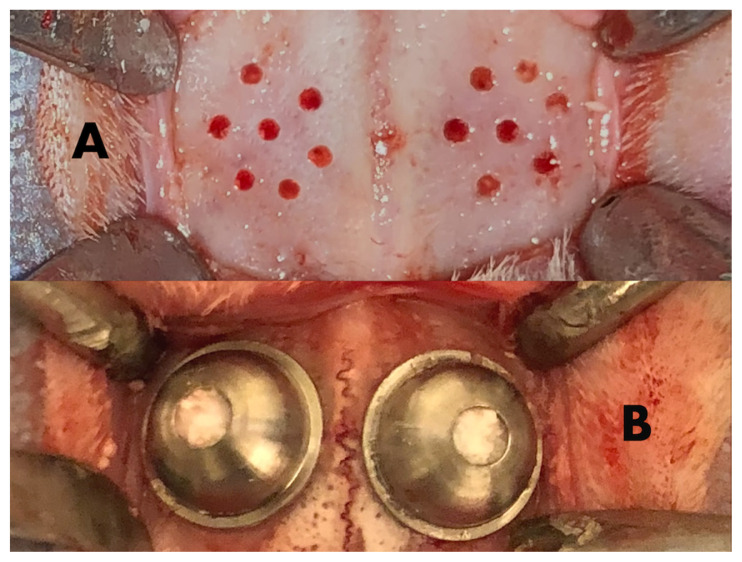
Protocol for three-dimensional reconstruction in a rabbit model. A. Decortication sites were opened up on the parietal bones. B. The area under the titanium cap (10 × 5 × 0.3 mm) was filled with a xenograft on each side and the left one was treated RSV.

All the animals were intramuscularly administered 35 mg/kg of ketamine hydrochloride (Alfamine, Alfasan, Woerden, The Netherlands) and 2.5 mg/kg of xylazine hydrochloride (Alfazyne, Alfasan, Woerden, The Netherlands) to induce anesthesia. The right and left cranial regions were shaved, and the operation site was disinfected using povidone-iodine (Batticon, Adeka, Samsun, Turkey). To control bleeding, the site was infiltrated using 1 mL of a local anesthetic solution (Ultracain D-S forte, Sanofi Aventis, Kırklareli, Turkey). Using a scalpel number 15, a ~4-cm long full-thickness incision was made on the calvarium midline along the linea media in order to include the periosteum, and the bone surface was exposed. Seven decortication sites were opened up on the parietal bones under sterile saline cooling using a round bur with a diameter of 1.5 mm (Figure 1A). A preprepared 7-hole titanium guide was used to standardize the decortication sites. Subsequently, specially prepared titanium caps with an outer diameter of 10 mm, a height of 5 mm, and a width of 0.3 mm were glued onto the decorticated bone using tissue adhesive (Leukosan Adhesive, Hamburg, Germany).

- In the RSV group, a 0.5–1 mm particle size 0.15 cc xenograft (OsteoBiol-Apatos, Giaveno, Italy) was soaked in a preprepared RSV solution (1 mg RSV-Sigma Aldrich-/0.4 mL sterile saline) and was placed into an opening with a diameter of 3 mm on the titanium cap.
- In the C group, 0.15 cc xenograft (OsteoBiol-Apatos, Giaveno, Italy) with a particle size of 0.5–1 mm was soaked in sterile saline was placed in the titanium cap using the same method (Figure 1B).

Finally, the skin and subcutaneous tissue were primarily sutured using a resorbable 16 mm 3/8 sharp 4.0 polyglactin suture (Coated Vicryl, Ethicon, Johnson & Johnson, Machelen, Belgium). Wound closure spray (Opsite, Smith & Nephew, Hull, UK) was applied onto the sutured regions to prevent postoperative infection.

During the postoperative period, the subjects were administered 1 mg/kg of meloxicam (Maxicam X4, Sanovel, İstanbul, Turkey) to induce analgesia and 2.5 mg/kg of intramuscular enrofloxacin as antibiotic therapy for 5 d (Baytril-K %5, Bayer, Kansas, US). The animals were kept alive in separate cages during the experiment under a light/dark cycle of 12 h each. The environmental temperature was maintained at an average of 22–24 °C, and the humidity, at 55%–70%. The health of the scar regions was regularly examined, and adequate amounts of food and water were supplied.

### 2.3. Tissue processing

The animals were sacrificed with a lethal dose of intramuscular xylazine hydrochloride (30 mg/kg Alfazyne, Alfasan, Woerden, The Netherlands) and ketamine hydrochloride (70 mg/kg Alfamine, Alfasan, Woerden, The Netherlands), half at the end of week 6 and the other half at the end of week 12. The study area was excised from each rabbit by an en bloc resection keeping some of the bone tissue intact around it. After the titanium caps were separated from the bone, the specimens were divided into subgroups in the case of each rabbit and were set in 10% buffered formaldehyde for 48 h.

### 2.4. Radiological analysis

Radiologic analysis was performed by an examiner who was blinded to the source of the samples. The specimens were scanned in a microcomputed tomography (CT) scanner (Skyscan 1174, Skyscan, Kontich, Belgium) with a pixel size of 40 μm. The x-ray tube voltage was 50 kV, and the current level was 800 μA. The exposure time was 2300 ms. X-ray projections were obtained at 0.70° intervals with a scanning angular rotation of 180°. Subsequent reconstructions of the raw data acquired during this screening phase were obtained using the software program, NRecon version 1.6.9.4 (NRecon, Skyscan, Kontich, Belgium). Subsequently, 8-bit gray images reconstructed using NRecon were transferred to the CTan software program (version 1.13.5.1, Skyscan, Kontich, Belgium). Basic values including bone volume, bone surface area, soft tissue volume, soft tissue surface area, bone/soft tissue ratio, bone density, etc. were obtained from selected regions of interest (ROI).

### 2.5. Histologic analysis

**Figure 2:**
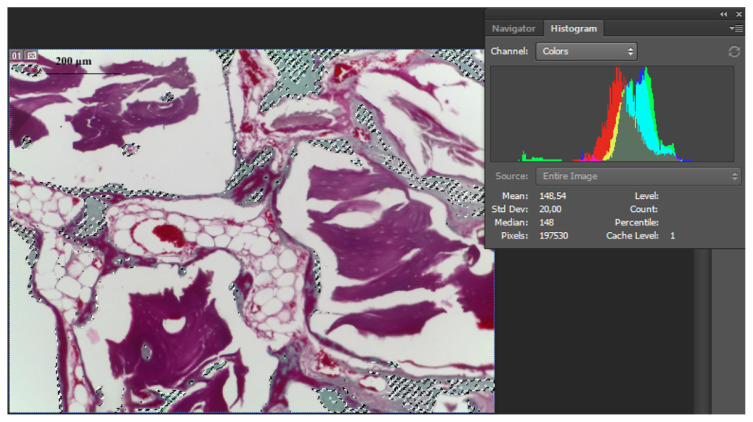
Console used to calculate the new bone trabecule/bone graft proportion by histogram method in Masson trichrome stained and ×10 magnified sample pictures.

Histological evaluations were performed in Hacettepe University, Faculty of Medicine, Department of Histology and Embryology. A histologic analysis was performed by an examiner who was blinded to the source of the samples. A total of 16 calvaria samples with two scaffolds on each were fixed in 10% formaldehyde for 48–72 h, and they were then decalcified in DeCastro solution (300 mL absolute ethanol, 50 g chloral hydrate, 670 mL distilled water, 30 mL 70% nitric acid) for 20 ± 2 d. After embedding the samples into paraffin blocks (Leica EC Embedding Center, Nussloch, Germany), they were sectioned into two halves along the midline of the scaffolds. Subsequently, 5 μm slices of the sections along the midline of each sample were prepared for the histological examination. The slices were deparaffinized in a 65° oven for approximately 2 h before the staining process. After processing them with xylol and serial alcohol for 30 min, respectively, the slices were stained with standard hematoxylin-eosin (HE) stain for the histopathologic evaluation. The samples were also stained with Masson’s trichrome stain to evaluate the degree of new bone formation. For Masson’s trichrome staining, the samples were treated with xylol and serial alcohol for 30 min, washed with distilled water, and kept in Bouin solution (picric acid 75 cc, 37%–40% formaldehyde 25 cc, glacial acetic acid 5 cc) in a 56° oven for 1 h. After the samples were washed in a stream of distilled water, they were stained with hematoxylin, washed again, and passed through acid-alcohol and ammonia water, respectively. Then, they were stained with trichrome dye (chromotrope 2R 0.6 g, light green 0.3 g, glacial acetic acid 1 cc, phosphotungstic acid 0.8 g, distilled water 100 cc) for 20 min, differentiated in acetic acid, and dehydrated by passing through serial alcohol. After staining, the samples were examined under a Leica DMR microscope (Leica Microsystems GmbH, Wetzlar, Germany) and were photographed under magnifications of ×4, ×10, and ×40 using a Leica Application System (LAS Version 4.2.0, Leica Microsystems GmbH, Wetzlar, Germany) connected to a personal computer. The digital images were evaluated for bone marrow formation, fibrous tissue formation, bone trabeculae formation, and bone scaffold formation during the bone healing process. In order to see the largest diameter of the defect, the sections were all cut along the midline. To objectively compare the morphological differences, the authors used a semiquantitative scoring system similar to the scale published by Santic et al., which allows for a statistical analysis of the differences in the bone healing processes between two groups of samples [13]. The evaluation was carried out by two independent observers. Bone marrow and fibrous tissue formation in the scaffold were graded on a three-point scale (grade 1 = located on lower 1/3 of the scaffold; grade 2 = reaches 2/3 of the scaffold; grade 3 = reaches the top of the scaffold) and new bone trabeculae count was evaluated on a three-point scale (grade 1, mild=≤5 new bone trabeculae; grade 2, moderate=6–10 new bone trabeculae; grade 3, intensive=≥11 new bone trabeculae). All the sections were evaluated with a magnification of ×10 and stained with H&E. While evaluating new bone formation, we measured quantitatively new bone trabeculae proportion to the bone graft particles in the scaffold areas. These calculations were displayed with ×10 magnification lenses in the samples stained with Masson trichrome dye. The newly formed bone trabeculae and the old bone tissue together with the graft in the defect area were selected separately by the histogram method (Adobe Photoshop CS6, California, USA) in different colors (new bone trabeculae in green, old bone tissue in red) (Figure 2). The calculation was performed in three different images of each sample at the midline sections. Following this, these values were summed up again and they were divided by the number of samples to determine the average values of each group.

Quantitative morphometric analysis of undecalcified new bone tissue was also calculated by micro CT which is a newer method than decalcified histomorphometry. Also we had compared the results of the two methods.

### 2.6. Statistical analysis

The data were analyzed using the SPSS 24.0 (SPSS Inc., Chicago, IL) statistical software package. Descriptive statistics (mean, median, standard deviation) were used in the analyses, and the Mann–Whitney U test was used to compare variables between the different groups. In each group, the data collected in the 6th week and 12th week were compared by the Wilcoxon test. Type 1-error level (alpha) values that were lower than 0.05 were considered statistically significant. The frequencies of the categorical data were presented as percentage values. Based on these results, the suggested sample size for each technique was calculated to run Wilcoxon–Mann–Whitney test two groups with a desired power = 0.80 and α = 0.05. The methodology was reviewed by an independent statistician.

## 3. Results

In all the groups, histopathological evaluations were performed at magnifications of ×4, ×10, and ×20. Scores were assigned to evaluate bone healing via standard HE staining, and the amount of bone trabeculae formed was measured by Masson’s trichrome staining. Bone trabeculae at various stages were observed in all the samples.

**Figure 3:**
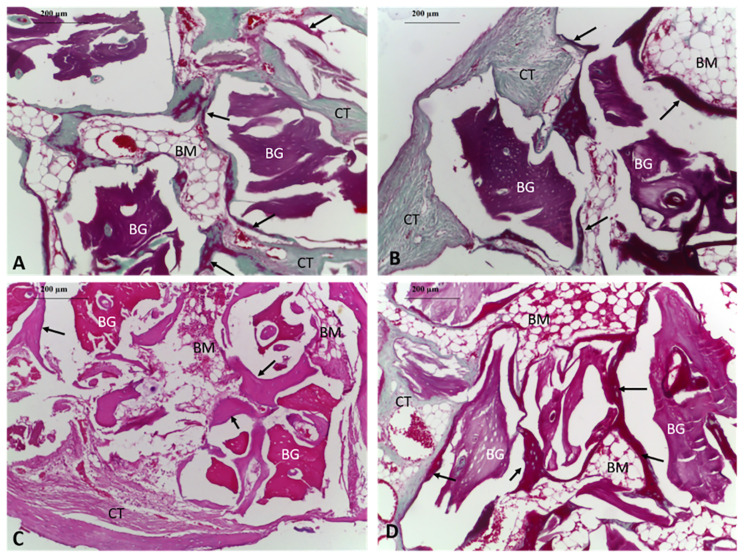
Histological findings of the defect area, 6 weeks (A, B) and 12 weeks (C, D) after surgery. A. In the group C defect area consists of anastomosing new bone trabeculae and loose collagen connective tissue (Masson trichrome, ×10). B. New bone trabeculae observed at the RSV group. (Masson trichrome, ×10). C. Group C showing anastomosing new bone trabeculae (less than in the RSV group), graft particles, and cellular structured collagen connective tissue (H&E, ×10). D. Specimens in the RSV group showing almost all bone graft particles surrounded by new bone trabeculae. (H&E, ×10). (H&E, hematoxylin and eosin; scale bar, 200 μm; group C, xenogeny bone grafting; RSV group, rosuvastatin with xenogeny bone grafting; BT, arrow, new bone trabeculae; BG, graft material; BM, bone marrow; CT, connective tissue).

The number and thickness of new bone trabeculae that were formed around the bone grafts in the RSV group were lower during the early period and increased during the late period. There were no differences in this group in terms of the distribution and extent of bone marrow and fibrous tissue formation. The C group was observed to exhibit less bone formation during the early period (Figure 3A). There were no changes in the newly formed bone trabeculae between weeks 6–12. Further, there were no differences in this group in terms of the distribution of bone marrow and connective tissue.

When the samples prepared in the 6th week were evaluated, it was observed that the bone height and density values of the new bone trabeculae in the C group were lower compared to those in the RSV group. No differences were observed between the groups in terms of the extent of connective tissue and bone marrow formation (Figure 3B). Examining the samples from the 12th week revealed that the RSV group exhibited superior results in terms of new bone trabeculae and total bone structure formation with the proportion of new bone being 0.3351 (Table 1). It was observed that almost all the bone grafts in the RSV group were surrounded by new bone tissue. (Figure 3C). No differences were observed between the groups in the connective tissue stage. Bone marrow development was also noted to be more prominent in the RSV group (Figure 3D, Table 2).

**Figure 4:**
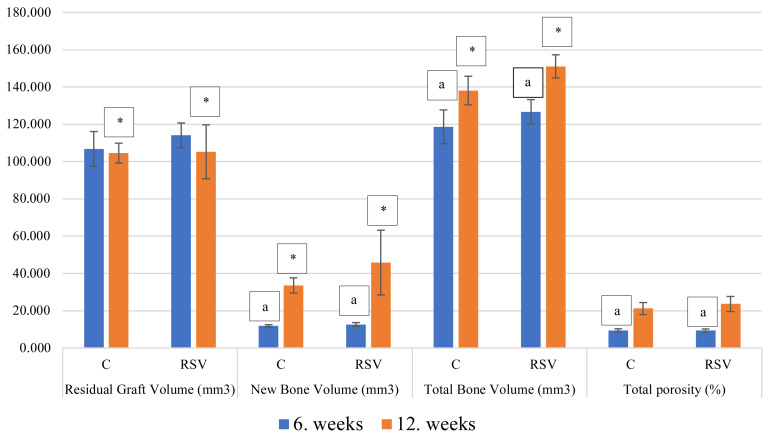
Comparison of values for the total bone volume, new bone volume, residual graft volume and total porosity derived from radiographs obtained 6 and 12 weeks after surgery. Data are presented as the mean ± standard deviation (Group C, xenogeny bone grafting only; group RSV, rosuvastatin with xenogeny bone grafting; a, significantly different from values obtained 12 weeks after surgery; *, significantly different from values obtained in the other group, black line indicates the standard deviation).

**Figure 5:**
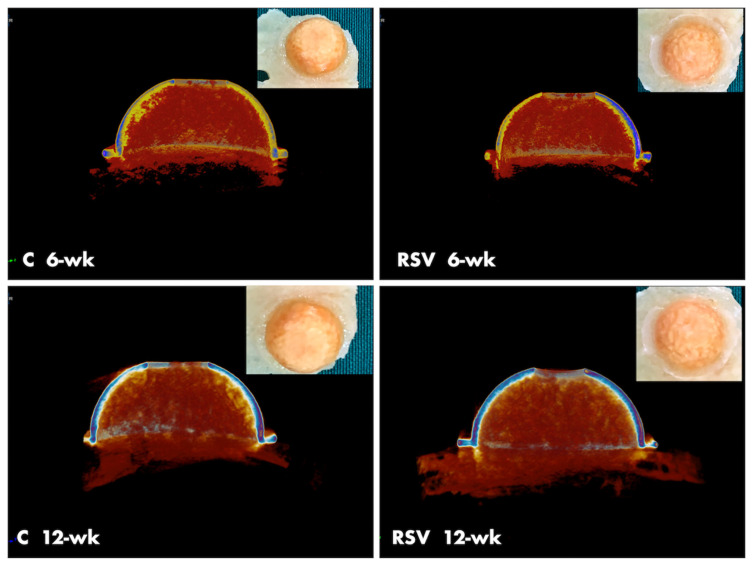
Microcomputed tomography (CT) images of all groups at 6 weeks and 12 weeks.

The radiological examination revealed that the total bone volume in the RSV group was significantly higher compared to in the C group at the 12-week timepoint (p < 0.05). In both the groups, the values in week 6 were significantly different compared to the values in week 12 (p < 0.05). The new bone volume was not significantly different between the groups in week 6 (p > 0.05), but in week 12, the values of the RSV group were significantly higher compared to those of the C group (p < 0.05). Additionally, at the 12-week timepoint, the values were significantly higher compared to those at the 6-week timepoint in both groups (p < 0.05). The residual graft volume values of the RSV group were significantly different compared to those of the C group at the 12-week timepoint (p < 0.05). There were no significant differences in values at the 6-week timepoint (p > 0.05). The bone density was not significantly different between the groups in both the weeks (p > 0.05). The total porosity (percent) and trabecular thickness values were not significantly different between the groups at both the timepoints (p > 0.05); however, the values at the 6-week timepoint were significantly different compared to those at the 12-week timepoint (p < 0.05), (Figures 4 and 5).

## 4. Discussion

The basis of the GBR approach is to prevent the invasion of fast-growing tissues into the bone regeneration site and to optimize bone healing. In addition, this approach protects the regeneration site and ensures that the relevant growth factors remain within the region. It is a method that has been used for a long time by many surgeons in the following procedures: vertical and horizontal augmentation of the alveolar bone, maxillary sinus lifting operations, and peripheral nerve surgeries. Today, jaw bones can be successfully reconstructed by maxillofacial surgeons using various GBR methods and materials [14,15].

The use of bone graft materials is very important in the three-dimensional reconstruction of bone defects. Autogenous grafts stimulate new bone formation owing to their osteogenic properties. Thus, they are the gold standard and are the primary choice when selecting bone graft material. However, since they require a second operation site, they increase surgery time, morbidity rates, postoperative discomfort, recovery time, and costs. Moreover, the rate of resorption of autologous grafts is still a serious disadvantage. Therefore, in clinical practice, it has become more common to use other graft materials, one of which is bovine bone grafts. Xenografts, such as bovine grafts, save the patient from autologous bone retention, provide a stable scaffold for bone formation, and maintain long-term graft volume stability owing to their low resorption rates [16]. These are the reasons why bovine bone grafts were preferred in this study. Xenografts could be found in different physical forms such as putty, granule or block. The physical characteristics of the grafts that affect bone healing through their osteoconduction properties are the pore size, the grain size, surface morphology and crystallinity of the material. Pores larger than 50 μm favor osteogenesis because of vascularization with cell recruitment and interconnecting pores of graft are beneficial for bone formation. In this study, a granule-shaped xenograft with a particle size of 0.5–1 mm, known to be the closest to the natural bone structure, was used. However, the results obtained in this study should be compared with the studies to be done with xenografts with different physical chemical structures [17].

Recently, it has been considered that the membrane used for GBR is more than just a passive barrier and that it plays an active role in promoting bone regeneration. Irrespective of the type of graft material used, ensuring stabilization through the duration of the healing period of the graft material is an important criterion for success. In addition, any mechanical stress applied onto the healing bone graft will cause the initial fibrin clot to deteriorate, leading to fibrous tissue being formed during the healing process. It is important to choose the most suitable type of surgical procedure from those involving the use of materials, such as collagen-containing membranes, titanium meshes, titanium caps, or bone screws. Collagen structured membranes are known to function as a mechanical barrier during the critical period that is specified as 3–4 weeks in the literature to allow for selective cell passage and to prevent inflammatory response during resorption [18]. However, these techniques cannot ensure sufficient stabilization of the graft material, especially in defects with fewer walls. To overcome this, we preferred to use rigid hemispheric titanium barriers in this study. It has previously been suggested that occlusive membranes can prevent certain types of growth factors from entering the site and, therefore, prevent bone formation [19,20]. However, subsequent studies have revealed that it is not required for undifferentiated mesenchymal cells to invade the site from surrounding soft tissues to ensure successful ossification in the region [21,22]. In this study, we observed that the use of a barrier without a pore structure did not affect bone formation. The most important advantages of hemispheric titanium barriers are their biocompatible structure and the ability to ensure proper stability owing to their adequate stiffness, especially in cases in which a particulate graft material is preferred; however, their disadvantages, such as mucosal perforations and the need for a second surgery, should also be considered.

The anabolic effects of statins on bone tissue were first described by Mundy et al., and studies on their effects on catabolic bone diseases, such as osteoporosis, have since been conducted [6]. In vitro studies have shown that statins affect osteoblast and osteoclast functions via the mevalonate pathway and that they have strong anabolic effects on vascular endothelial cells and endothelial progenitor cells. While in vivo animal studies have demonstrated that statins administered systemically can reduce bone loss due to inflammation, some clinical studies have reported that they can reduce the risk of osteoporotic bone fracture and increase bone mineral density [23]. Previous studies have also demonstrated that statins exhibit low bioavailability when they are orally administered, since they are rapidly absorbed and metabolized in the liver [24]. Thus, as the administration of statins gained popularity, local application methods started to be investigated, and it was shown that statins applied topically were 50 times more active than those applied orally [25]. There have since been many animal studies that involved RSV being applied locally in fracture healing, implant applications, long bone defect models, and craniofacial defect models. There are also several clinical trials in which RSV was applied locally, especially in patients with periodontal diseases. In light of this information, it was considered that local application was more effective in optimizing the osteoinductive effect; therefore, local application was preferred in this study.

It is noteworthy to mention that there are a variety of methods outlined in the literature via which statins can be applied locally. Wu et al. used polylactic acid/polyglycolic acid copolymers as carriers, and Ibrahim et al. transferred statins to the relevant site using a biodegradable sponge [26,27]. Mukozawa et al. used a hydrogel and absorbable collagen sponge as the carrier material and achieved similar positive results with both [28]. Liu et al. used a hydrogel as the carrier for the intraosseous application of statins and highlighted the positive features of hydrogels, such as biocompatibility, low toxicity, and adequate stability [29]. Monjo et al. mentioned that the sustained release of a compound with low molecular weight, such as statins, would not be possible with the use of any carrier system [10]. However, there is no clear information in the literature on which carrier system is more advantageous. In this study, the bone graft material was also used as the carrier material. Therefore, no other material was required to act as a carrier. The results of this study also support that an additional carrier material is not required for statin application and that the graft material can also serve as a good carrier. We considered that one reason for this is that the barrier we used is of the rigid occlusive type. In future studies, a suitable carrier system to improve the slow release of statins, to facilitate bone formation, and to prevent the initial inflammatory response should be investigated.

RSV, a HMG-CoA reductase inhibitor, produces the strongest effect among the currently used statins and is effective in lowering low-density lipoprotein (LDL) cholesterol levels [30]. According to Karlson et al., RSV is eight times stronger than simvastatin [9]. In addition, Pradeep et al. demonstrated that RSV was a more effective type of statin in their study in which they compared the effects of RSV with those of atorvastatin [31]. RSV was chosen as the statin type in this study, and positive effects on bone graft healing were demonstrated.

During our literature review, we did not find a definite consensus on the appropriate dose of locally administered RSV. Pradeep et al. used 1.2 mg of RSV for intrabone defects and furcation defects in their study [31,32]. Monjo et al. used three different doses, 0.1, 0.5 and 2.5 mg, and outlined the toxic effects that may occur in the case of higher doses [10]. Türer et al. used two different doses, 0.1 and 1 mg, in their calvarial bone defect study on rats and determined that the optimal dose was 1 mg [33]. In their subsequent study, they used a fractured rat mandible model and a 1 mg dose of RSV and demonstrated a positive effect on healing [34]. In the current study, a 1 mg dose of RSV was used, and it was demonstrated to have positive effects on both new bone and total bone volume.

The reason why rabbits were chosen as the experimental animals in this study was to ensure that both the study sample groups were from the same animal in order to prevent individual differences. We decided to use the calvaria as our study site for the following reasons: it is not difficult to work on this site; complications, such as, postoperative exposures were less likely to occur in this region; and we considered the study models used in the literature [21,22]. According to Ito et al., titanium caps have been evaluated to be an important material that results in bone formation in regions without bones [35]. In the study by Marechal et al., occlusive titanium barriers that were used for bone augmentation were reported to be more advantageous than other techniques with respect to new bone formation [36]. Unlike in the case of a defect model, titanium caps that have been used by many researchers to induce bone formation in regions with no bone tissue were observed to yield successful results in this study. The key points in achieving these successful results are ensuring the stabilization of the barrier material and impermeability between the bone and barrier. In this study, the stabilization and impermeability of the titanium caps were facilitated by N-butyl-2-cyanoacrylate that is often used as a tissue adhesive. Moreover, known to play an important role in bone augmentations, decortication has been demonstrated to be indispensable in ensuring blood supply and increasing bone morphogenetic protein and other growth factor levels in previous studies [36–38]. Furthermore, Min et al., in their study on rabbits, demonstrated that decortication was crucial; however, the size of the decortication site was insignificant in terms of augmentation. Therefore, in this study, decortication was performed in the regeneration area under the titanium cap, and it was ensured that the site was equal in size in all the groups to ensure standardization.

In previous studies on rabbits, a 4-week period was found to be sufficient to ensure angiogenesis and new bone formation in the defect region [39]. However, unlike in the case of the defect model, this study dealt with bone formation in an area with no prior bone. On researching studies that involved similar models and histological and radiological changes, we found that a 12-week period was required to determine the bone regeneration capacity of RSV [24,25]. In this study, the study timepoints were determined to be 6 and 12 weeks to examine the early and late effects of RSV on bone augmentation.

The most reliable methods to measure newly formed bone and soft tissue amounts are two-dimensional histomorphometric analyses and three-dimensional micro-CT analyses. However, in histomorphometric examinations, larger sections of samples are required compared to in a micro-CT analysis. Therefore, this technique has been reported to generate more accurate and detailed results compared to in studies that used micro-CT analyses [40]. However, the samples used in micro-CT analyses do not get degraded, which is very important as it allows for histological examinations and comparisons. In this case, the histological results were supported by the radiological results.

The effects of RSV have previously been investigated in various models, such as in defect and fracture models; however, it was used in a three-dimensional reconstruction model for the first time in this study, and its positive effects on bone healing were demonstrated. We consider that such methods that can result in bone formation in regions with no prior bone tissue unlike in defect model studies, would be highly effective in procedures, such as vertical and horizontal alveolar bone reconstructions, which involve challenges and requirements, such as implant surgery, before they are performed.

Bone regeneration is a complex, well-orchestrated physiological process of bone formation, which can be seen during normal bone healing, and is involved in continuous remodeling throughout adult life. In this study, it has been shown that RSV demonstrates its efficacy on new bone formation and total bone volume. This study includes a 12-week recovery period, and the effectiveness of RSV in this process has been comparatively demonstrated over two different time periods. Therefore, instead of using the term bone healing, we used the term bone formation in our hypothesis. In addition, for bone healing, resorption of all graft material and new bone formation must be completed. In this study, using the term bone healing was avoided. The total bone volume data included both new bone and residual graft amount. When analyzing the results of this study, new bone formation data will be more valuable than the total bone volume data. Thus new bone formation that defines bone formation can be carefully studied.

Finally, based on the results of this study, the statin, RSV, when administered locally produces strong effects that stimulate bone formation. However, further studies are needed with different doses and graft materials using different animal models.

## Figures and Tables

**Table 1:** The new bone trabeculae/bone graft proportion of the samples.

	Groups	New bone proportion
6 weeks	RSV (n = 4)	0.1320 ± 0.0579
	C (n = 4)	0.0937 ± 0.0227
12 weeks	RSV (n = 4)	0.3351 ± 0.0156
	C (n = 4)	0.1975 ± 0.0493

**Table 2:** The frequency of histomorphological parameters as a percentage.

	Groups	Bone marrow grade (%)			New bone on the surface grade (%)			New bone trabeculae grade (%)			Fibrous tissue formation grade (%)		
		1	2	3	1	2	3	1	2	3	1	2	3
6 weeks	RSV	-	75	25	-	25	75	25	75	-	100	-	-
	C	25	75	-	25	62.5	12.5	75	25	-	75	12.5	12.5
12 weeks	RSV	12.5	37.5	50	-	12.5	87.5	12.5	75	12.5	100	-	-
	C	-	87.5	12.5	-	50	50	75	25	-	75	25	-
